# Post-annealing Effect on Optical and Electronic Properties of Thermally Evaporated MoO_X_ Thin Films as Hole-Selective Contacts for *p*-Si Solar Cells

**DOI:** 10.1186/s11671-021-03544-9

**Published:** 2021-05-19

**Authors:** Yuanwei Jiang, Shuangying Cao, Linfeng Lu, Guanlin Du, Yinyue Lin, Jilei Wang, Liyou Yang, Wenqing Zhu, Dongdong Li

**Affiliations:** 1 School of Materials Science and Engineering, Shanghai University, 149 Yanchang Road, Jing’an, Shanghai, 200072 China; 2 CAS Key Lab of Low-Carbon Conversion Science and Engineering, The Interdisciplinary Research Center, Shanghai Advanced Research Institute, Chinese Academy of Sciences, 99 Haike Road, Zhangjiang Hi-Tech Park, Pudong, Shanghai, 201210 China; 3 Jinneng Clean Energy Technology LTD, 533 Guang’an Street, Jinzhong, 030600 China; 4 University of Chinese Academy of Sciences, 19 Yuquan Road, Beijing, 100049 China

**Keywords:** Silicon heterojunction solar cells, MoO_X_ hole-selective contacts, Hole selectivity, Work function, Optoelectronic properties

## Abstract

Owing to its large work function, MoO_X_ has been widely used for hole-selective contact in both thin film and crystalline silicon solar cells. In this work, thermally evaporated MoO_X_ films are employed on the rear sides of *p*-type crystalline silicon (*p*-Si) solar cells, where the optical and electronic properties of the MoO_X_ films as well as the corresponding device performances are investigated as a function of post-annealing treatment. The MoO_X_ film annealed at 100 °C shows the highest work function and proves the best hole selectivity based on the results of energy band simulation and contact resistivity measurements. The full rear *p*-Si/MoO_X_/Ag-contacted solar cells demonstrate the best performance with an efficiency of 19.19%, which is the result of the combined influence of MoO_X_’s hole selectivity and passivation ability.

**Supplementary Information:**

The online version contains supplementary material available at 10.1186/s11671-021-03544-9.

## Introduction

Transition metal oxides possess a wide range of work functions, spanning from 3.5 eV for defective ZrO_2_ to 7.0 eV for stoichiometric V_2_O_5_ [1–6]. Among them, MoO_X_ is one of the most extensively studied materials for applications in optoelectronic devices [7–9] due to its high transparency, nontoxicity and moderate evaporation temperature [10, 11]. MoO_X_ is reported to have a large work function of ~ 6.7 eV and is being widely used as hole extraction layers in photovoltaic devices [12], light emitting devices [13], sensors [14, 15] and memories [16]. For photoelectric devices involving MoO_X_ hole extraction layers, the device performance is strongly dependent on both the optical and electronic properties of the MoO_X_ thin films. In the photovoltaic field, MoO_X_ thin films were initially applied in organic devices [17–19]. In recent years, a lot of research has been done on the application of MoO_X_ films to crystalline silicon (*c*-Si) solar cells [9, 20–22]. The ionization energy of *c*-Si is about 5.17 eV, which is the lower limit for the work function of hole selective contact materials [23]. The high work function of MoO_X_ will induce a large band bending at the *c*-Si/MoO_X_ interface and lead to the accumulation of holes in *p*-type silicon (*p*-Si) or the depletion of electrons in *n*-type silicon (*n*-Si), thus favoring the holes transport [24]. By substituting the *p*-type amorphous silicon layer with MoO_X_ film in the classical silicon heterojunction solar cell, an power conversion efficiency (*PCE*) of 23.5% has been achieved [25]. Compared to MoO_X_ contacts made to *n*-type wafers, those made to *p*-type wafers (without amorphous Si layer) show better performance in terms of surface passivation and contact resistivity [24]. The feasibility of MoO_X_ films as hole-selective contacts on *p*-Si solar cells has been demonstrated in our previous work [26], and an efficiency of 20.0% was achieved based on *p*-Si/SiO_X_/MoO_X_/V_2_O_X_/ITO/Ag rear contact [27].

MoO_X_ (X ≤ 3) has a large work function because of the closed shell character in its bulk electronic structure and the dipoles created by its internal layer structure [28]. The presence of oxygen vacancy defects will decrease the work function of MoO_X_ [4] and result in an *n*-type material [29]. Numerical simulations indicated that higher work function of MoO_X_ induced a favorable Schottky barrier height as well as an inversion at the MoO_X_/intrinsic a-Si:H/*n*-type *c*-Si (*n*-Si) interface, stimulating the path of least resistance for holes [30]. Therefore, tuning the electronic structure and work function of MoO_X_ is of great significance for passivating contact *c*-Si solar cells.

MoO_X_ films can be deposited by atomic layer deposition [30–34], reactive sputtering [12], pulsed laser deposition [35], thermal evaporation [24, 36] and spin coating [37]. In most of the solar cell researches based on Si/MoO_X_ contact, MoO_X_ films are prepared by thermal evaporation at room temperature [8]. Because the controllability of the properties of MoO_X_ films by thermal evaporation is limited, various methods of post-treatments were studied to tune the work function of thermally evaporated MoO_X_. UV-ozone exposure could increase the work function of evaporated MoO_X_ films on gold substrates from 5.7 eV to 6.6 eV [8]. Irfan et al. performed air annealing of MoO_X_ films on gold substrates at 300 °C for 20 h and found that the long-time annealing does not assist in reducing the oxygen vacancies due to the diffusion of gold from substrate toward the MoO_X_ film [38]. The work function of MoO_X_ films on *p*-type *c*-Si (*p*-Si) was found to decrease after *in situ* vacuum annealing in the temperature range from 300 to 900 K [39].

In this work, *p*-Si solar cells with MoO_X_ passivating contacts on rear sides are configured. The optical and electronic properties as well as the influence of the post-annealed MoO_X_ films on *p*-Si/MoO_X_ solar cells are systematically investigated through experiments and energy band simulations. A linear relationship between the work function and the O/Mo atomic ratio is found. It is interesting that compared with the intrinsic sample, the 100 °C-annealed sample with a higher work function exhibits a lower contact resistivity in spite of its thicker SiO_X_ interlayer. According to the energy band simulation, the variation of MoO_X_’s work function has a little effect on the band bending of *p*-Si, while the band bending of MoO_X_ increases significantly as its work function increases. Therefore, it is suggested that higher work functions are vital for effective hole transport from *p*-Si to MoO_X_ where the interfacial SiO_X_ layer is in a moderate thickness range. Our results provide valuable details of the interface characteristics of the *p*-Si/MoO_X_ in view of high-performance heterojunction solar cells with oxide-based carrier selective contacts.

## Methods

### Film Deposition, Post-Annealing Process and Solar Cell Fabrication

Solar cells are fabricated on *p*-type < 100 > CZ wafers with a resistivity of ~ 2 Ω·cm and wafer thickness of 170 μm. The silicon wafers are precleaned by mixed solution of NaOH and H_2_O_2_ and then textured by NaOH solution. The wafers are then washed by deionized water (DI water) following 1 min’s dip in dilute hydrofluoric acid (HF). Heavily doped n^+^ front surface (*N*_D _≈ 4 × 10^21^ cm^−3^) is achieved by diffusing phosphorus from a POCl_3_ source in a quartz furnace. A double-layered SiN_X_:H passivation and antireflection coating is then deposited by plasma-enhanced chemical vapor deposition (PECVD). The silver paste is screen-printed on the solar cells with a selective emitter [40]. Subsequently, a fire-through process is conducted at 850 °C for ~ 1 min, after which Ohmic contacts with low resistivity result [41]. The rear surface of each sample is rinsed with dilute HF before MoO_X_ deposition. MoO_X_ films are thermally evaporated at the rear side with a deposition rate of ~ 0.2 Å/s under 8 × 10^–4^ Pa [26]. Post-annealing treatments of the room-temperature-deposited MoO_X_ films are carried out in a rapid thermal processor in air. The setting temperature was reached in 10 s and held for 5 min. MoO_X_ films with different annealing temperatures are applied to *p*-Si solar cells with full rear MoO_X_/Ag contacts.

### Measurements

The transmittance spectra of the MoO_X_ films deposited on 1.2-mm-thick silica glasses are measured using a UV–Vis spectrometer with an integrating sphere. Surface morphology and roughness of the films are measured by atomic force microscope (AFM). The optical properties of the MoO_X_ films are analyzed using spectroscopic ellipsometry (J.A. Woollam Co., Inc., M2000U ellipsometer), and the measured results are fitted using the native oxide model. High-resolution X-ray photoelectron spectroscopy (XPS) of Mo 3d and Si 2p are measured employing monochromate Al Kα X-rays with a photon energy of 1486.7 eV. The ultraviolet photoemission spectroscopy (UPS) spectra are recorded by using unfiltered He I 21.22 eV excitation with the sample biased at − 10 eV. Before XPS and UPS detecting, the surfaces of the samples were precleaned by argon ions.

The contact resistivity at *p*-Si/MoO_X_ interface is extracted by the Cox and Stack method [42], which involves a series of resistance measurements on a probe station with different diameter front Ag contacts. The passivation qualities of MoO_X_ films with different thicknesses are determined from effective lifetime measurements via quasi-steady-state photo conductance (QSSPC) method. The samples for QSSPC test are asymmetric as the front sides are textured, *n*^+^ doped and passivated by means of a double-layered SiN_X_:H films [43], while the rear sides are covered with the MoO_X_ films [26]. The current density–voltage characteristics of the solar cells (3.12 × 3.12 cm^2^) are measured under standard one sun conditions (100 mW·cm^−2^, AM1.5G spectrum, 25 °C) as the luminous intensity is calibrated with a certified Fraunhofer CalLab reference cell.

### Simulations

Numerical simulation of the band structure of the *p*-Si/MoO_X_ contacts is done with AFORS-HET, which is based on solving the one-dimensional Poisson and two carrier continuity equations [44]. The key parameters are listed in Table 1. The front and back contact boundary is set as fixing metal work function to flat band. The interface between *p*-Si and MoO_X_ is set as “thermionic-emission” (one of the numerical models). Tunneling properties of thin SiO_2_ film are commonly set by changing the interface parameters under the “thermionic-emission” model only for metal/semiconductor Schottky contact. Therefore, the actually existed tunneling SiO_X_ at the Si/MoO_X_ interface is omitted. For *p*-Si, electroneutral defects at the central energy with total trap density is set as 1 × 10^14^ cm^−3^. For MoO_X_, donor-type conduction tail defects with total concentration are set as 1 × 10^14^ cm^−3^.

**Table 1:** Parameters used for AFORS-HET simulation

Parameters	*p*-Si	MoO_X_
Layer thickness (cm)	1 × 10^–4^	1 × 10^–6^
Doping concentration (cm^−3^)	1 × 10^16^ (acceptor)	1 × 10^16^–1 × 10^20^ (donor)
Relative dielectric constant	11.9	10
Electron affinity (eV)	4.05	6.2
Band gap (eV)	1.124	3.3
Effective conduction band density (cm^−3^)	2.843 × 10^19^	1 × 10^20^
Effective valence band density (cm^−3^)	2.682 × 10^19^	1 × 10^20^
Electron mobility (cm^2^/Vs) [45]	1107	30
Hole mobility (cm^2^/Vs) [45]	424.6	2.5

## Results and discussion

**Fig. 1:**
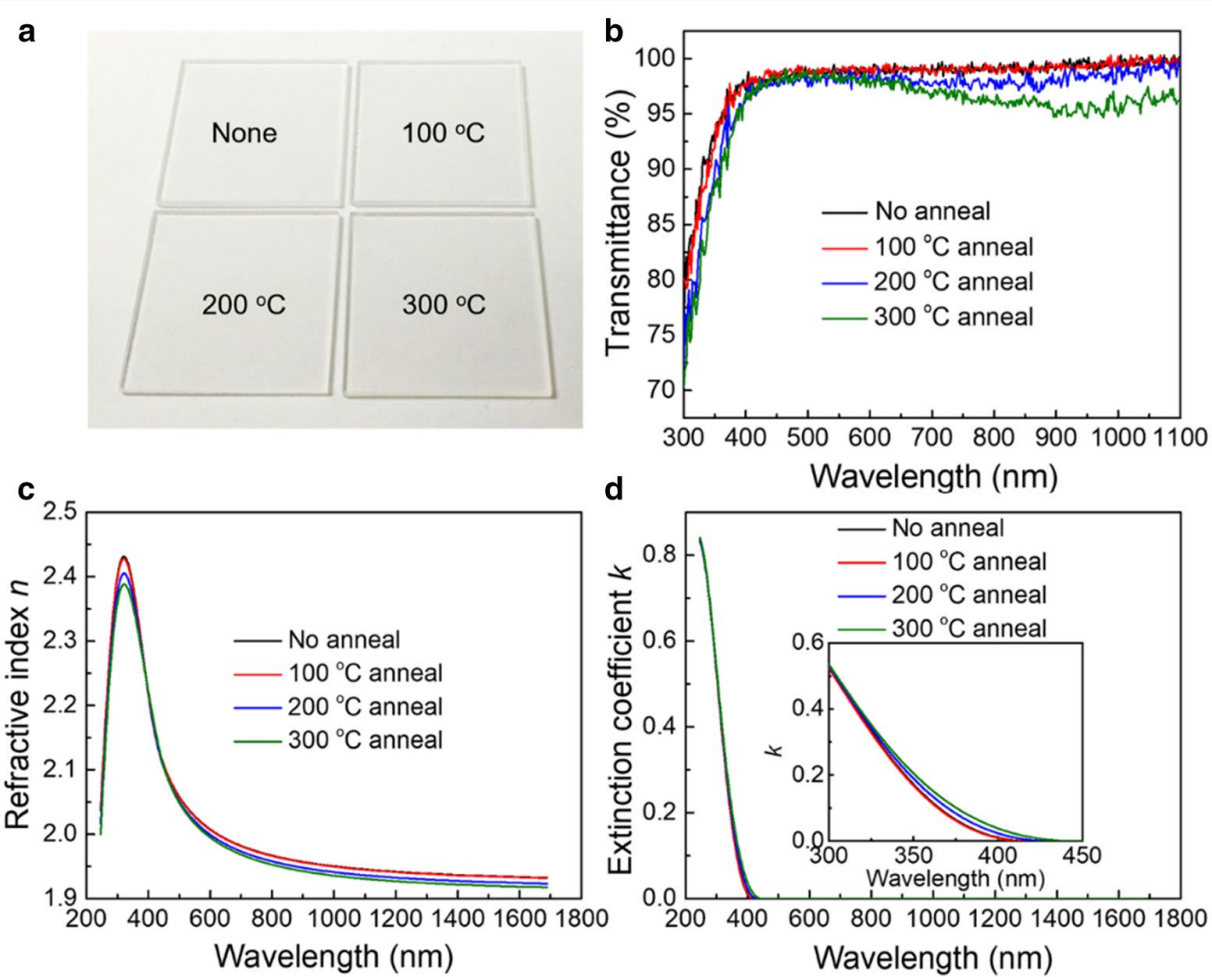
**a** Photographs and **b** transmittance spectra of the 10-nm-thick MoO_X_ films on silica glass annealed in air for 5 min at different temperatures. **c** Refractive indices *n* and **d** extinction coefficient *k* curves of the 20-nm-thick MoO_X_ films on polished silicon wafers

Figure 1a represents the photographs of the 10-nm-thick MoO_X_ films on silica glass annealed in air for 5 min at different temperatures (100 °C, 200 °C and 300 °C). All of the samples are visually colorless and transparent. From the corresponding optical transmittance spectra in Fig. 1b, one can see that the transmittance spectrum of the 100 °C-annealed MoO_X_ film almost overlaps with that of the unannealed film. Higher annealing temperatures result in a lower transmittance at 600–1100 nm range, which could be assigned to free carrier absorption induced by oxygen vacancies [46]. Thicker MoO_X_ films (20 nm) are deposited onto polished Si wafers to measure the refractive index *n* and extinction coefficient *k* more accurately. The refractive index in Fig. 1c lies in the 1.8–2.5 range, which is consistent with other studies [31, 32]. The *n* curves as well as the *k* curves (Fig. 1d) have a little difference among the four samples. The *n* at 633 nm of the 20-nm-thick films decreases slightly, which is summarized in Table 2.

**Table 2:** Root-mean-square roughness (unit: nm) of 10 nm/20 nm post-annealed MoO_X_ films on SiO_2_ wafers and refractive index *n* at 633 nm of the 20-nm films

Annealing temperature (°C)	None	100	200	300
RMS-10 nm	4.116	8.806	12.124	12.913
RMS-20 nm	1.399	0.940	0.845	0.709
*n* at 633 nm	1.998	1.997	1.989	1.984

The surface morphologies are then characterized by AFM as shown in Additional file 1: Figure S1. The corresponding root-mean-square (RMS) roughness is listed in Table 2. The as-deposited 10-nm-thick MoO_X_ thin film (Additional file 1: Figure S1a) has an RMS roughness of 4.116 nm, which is in accordance with the wave-like surface morphology. As the annealing temperature goes higher (Additional file 1: Figure S1b–d), the surface undulation of the MoO_X_ film becomes larger, while the featured structures become smaller and much denser probably due to the dewetting process [47]. After annealing at 300 °C, the RMS roughness reaches 12.913 nm. The 20-nm-thick films are less rough with the RMS around 1 nm (Table 2). The dewetting process is also suppressed as indicated by the RMS measurements as a function of annealing treatments. The above morphology evolution does not fully reflect the changes in the oxide film in the device level, where the MoO_X_ films are deposited on Si and capped with Ag electrodes, but the morphology evolution can do give us the intrinsic properties of MoO_X_ on SiO_2_ surface.

**Fig. 2:**
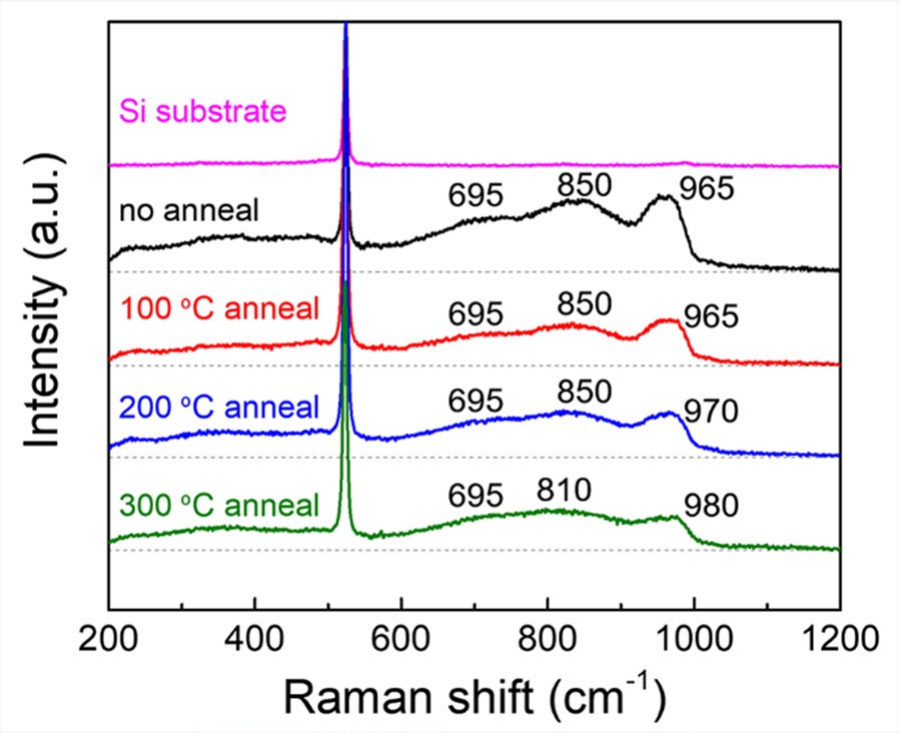
The UV Raman (325 nm) spectra of post-annealed 20-nm-thick MoO_X_ films on polished silicon wafers

MoO_X_ has a natural tendency to form oxygen vacancy defects [48], which may impact on the molecular structure. In order to identify such vacancy-related molecular structure variations, Raman spectroscopy measurements are taken on MoO_X_(20 nm)/Si(< 100 >). There are no characteristic peaks of MoO_X_ in the Raman spectra under green light (532 nm) excitation (Additional file 1: Figure S2), which is independent to the thermal treatment. When the excitation is changed to ultraviolet light of 325 nm, characteristic bands of MoO_X_ appear, which generally locate at 600–1000 cm^−1^ (Fig. 2). The sharp peak of 515 cm^−1^ in all samples corresponds to Si–Si bond. For the intrinsic and 100 °C-annealed MoO_X_ films, Raman bands are present at 695, 850 and 965 cm^−1^, which are from [Mo_7_O_24_]^6−^, [Mo_8_O_26_]^4−^ anions, and (O =)_2_Mo(–O–Si)_2_ dioxo species, respectively [49]. When the film is annealed at 200 °C, the 965 cm^−1^ band shifts to 970 cm^−1^, which is assigned to Mo(= ^16^O)_2_ dioxo species [50]. The Raman spectrum of the 300 °C-annealed MoO_X_ film exhibits bands at 695, 810 and 980 cm^−1^. The band at 810 cm^−1^ is from Si–O–Si bond, while the (O =)_2_Mo(–O–Si)_2_ contributes the band at 980 cm^−1^. The results indicate that annealing at different temperatures will affect the chemical composition of MoO_X_ film, which may indicate the difference of oxygen vacancy concentration of each sample.

**Fig. 3:**
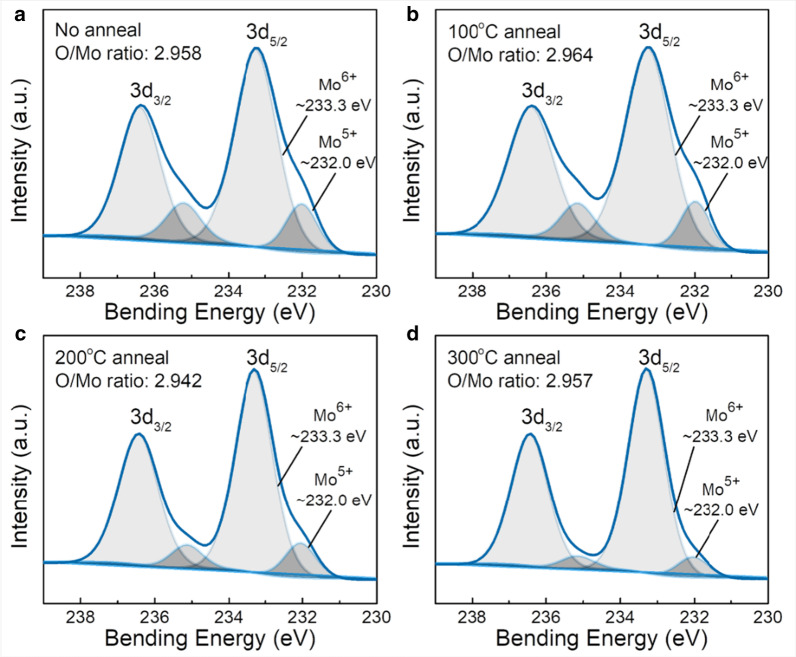
Mo 3d core-level XPS spectra of the 10-nm-thick MoO_X_ films on silicon wafers **a** without post-annealing, with post-annealing at **b** 100 °C, **c** 200 °C and **d** 300 °C

XPS is conducted on MoO_X_ films (10 nm) to quantify the relative content of each oxidation state and the oxygen to molybdenum (O/Mo) atomic ratios. After Shirley background subtraction and fitting by Gaussian–Lorentzian curves, a multi-peak deconvolution of the XPS spectra is conducted. The Mo 3d core level is decomposed into two doublet peaks with a doublet spin–orbit splitting *Δ*_BE_ 3.1 eV and a peak area ratio of 3:2 [11]. As shown in Fig. 3, the peak of Mo^6+^ 3d_5/2_ core level centers at ~ 233.3 eV binding energy. For all of the samples, a second doublet at ~ 232.0 eV, which is denoted as Mo^5+^, is required to obtain a good fit to the experimental data [8]. The O/Mo ratio is calculated by the following formula [51]:

$$X = \frac{1}{2} \cdot \frac{{\mathop \sum \nolimits_{n} n \cdot I({\text{Mo}}^{n + } )}}{{\mathop \sum \nolimits_{n} I({\text{Mo}}^{n + } )}}$$

where *I*(Mo^*n*+^) is the individual component intensities from the Mo 3d spectra. *n* relates to the valence state of Mo ion, i.e., 5 for Mo^5+^ and 6 for Mo^6+^. The factor 1/2 is due to that each oxygen atom is shared by two molybdenum atoms.

The O/Mo ratios of all samples as listed in Table 3 are below 3. Oxygen loss and oxidation state transitions have been reported during transition metal oxides deposition [1]. Since the XPS measurements are ex-situ, the air exposure to the thermally evaporated MoO_3_ films at room temperature could also increase the oxygen vacancies [18, 52]. The O/Mo ratio of the unannealed MoO_X_ film is 2.958, while post-annealing at 100 °C increases the value to 2.964. Higher annealing temperatures then reduce the O/Mo ratio gradually. The highest O/Mo ratio of the 100 °C-annealed sample might be explained by the thermally activated oxygen injected from air to the MoO_X_ film [38]. Additional file 1: Figure S3 compares the Si 2p XPS spectra of the 10-nm-thick annealed MoO_X_ films. The Si 2p XPS spectrum of the unannealed sample shows dual peaks of silicon elements and Si^4+^ peak. A Si^2+^ peak appears when annealed at 100 °C. When annealed at 200 and 300 °C, peaks of Si^4+^, Si^3+^ and Si^2+^ exist simultaneously. In addition, the calculated X in SiO_X_ for the four samples are 2, 1.715, 1.672 and 1.815, respectively. The oxygen atoms in SiO_X_ are from MoO_X_; therefore, the O/Mo ratio depends on the balance between SiOx taking oxygen and air injecting oxygen. By the way, as the annealing temperature goes higher, the signal of Si element becomes weaker, indicating thicker SiO_X_ interlayers [26].

**Table 3:** O/Mo ratio and work function of the post-annealed 10-nm-thick MoO_X_ films on silicon wafers. Effective minority carrier lifetime of silicon wafers covered by the post-annealed MoO_X_ films

Annealing temperature (°C)	None	100	200	300	Without MoO_X_ (bare Si)
O/Mo ratio	2.958	2.964	2.942	2.957	
Work function (eV)	6.24	6.27	6.21	6.25	
Effective minority carrier lifetime (μs)	26.70	21.53	15.41	9.44	7.76

**Fig. 4:**
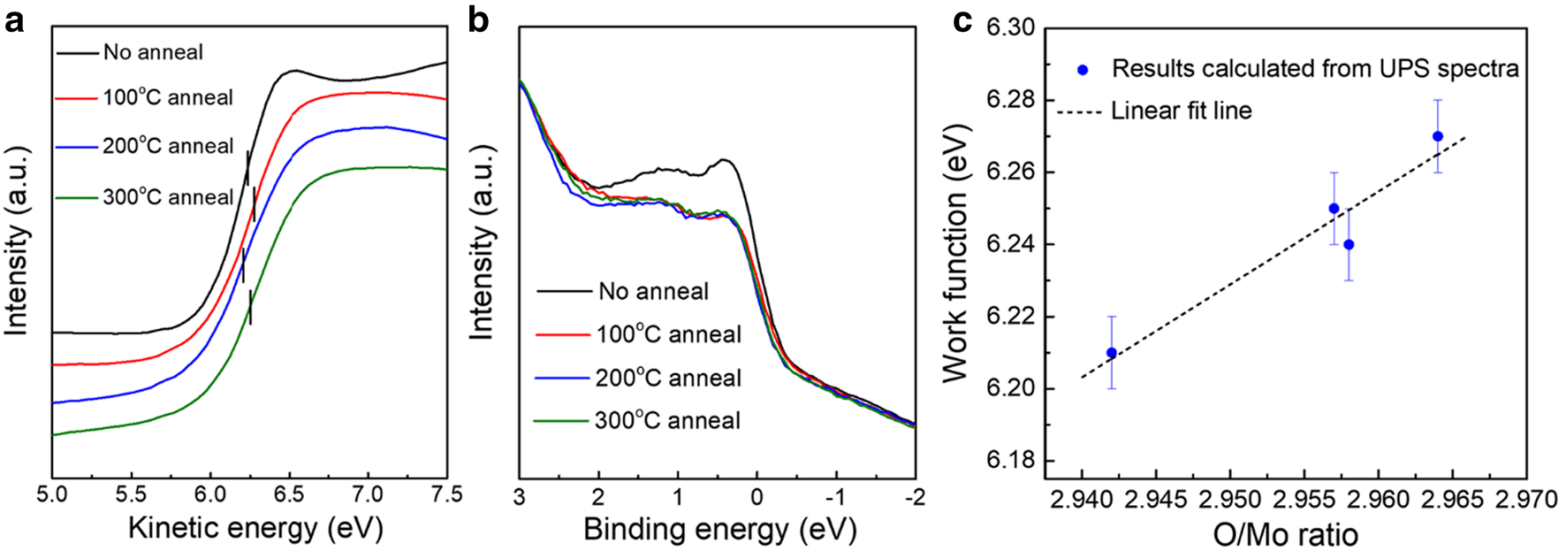
**a** The secondary electron cutoff region and **b** valence band from the UPS spectra of the post-annealed MoO_X_ films on silicon wafers. **c** Work function plotted against the stoichiometry (O/Mo ratio)

Reducing the cation oxidation state of an oxide tends to decrease its work function [1]. UPS is utilized to calculate the work function of MoO_X_ films as a function of thermal treatment. Figure 4a shows the secondary electron cutoff region of the UPS spectra, from which a minor vibration of work function can be seen. From Fig. 4b we can see, after post air annealing, the defect peaks in the valence band area [37] become weaker. Table 3 lists the O/Mo ratio evaluated from XPS fitting and corresponding work function evaluated from UPS secondary electron cutoff for samples on polished silicon wafers. The results of the work function and the stoichiometry of MoO_X_ are also depicted in Fig. 4c, where a strong positive correlation is disclosed. An increase of the O/Mo ratio from 2.942 to 2.964 leads to an increase of the work function by roughly 0.06 eV.

**Fig. 5:**
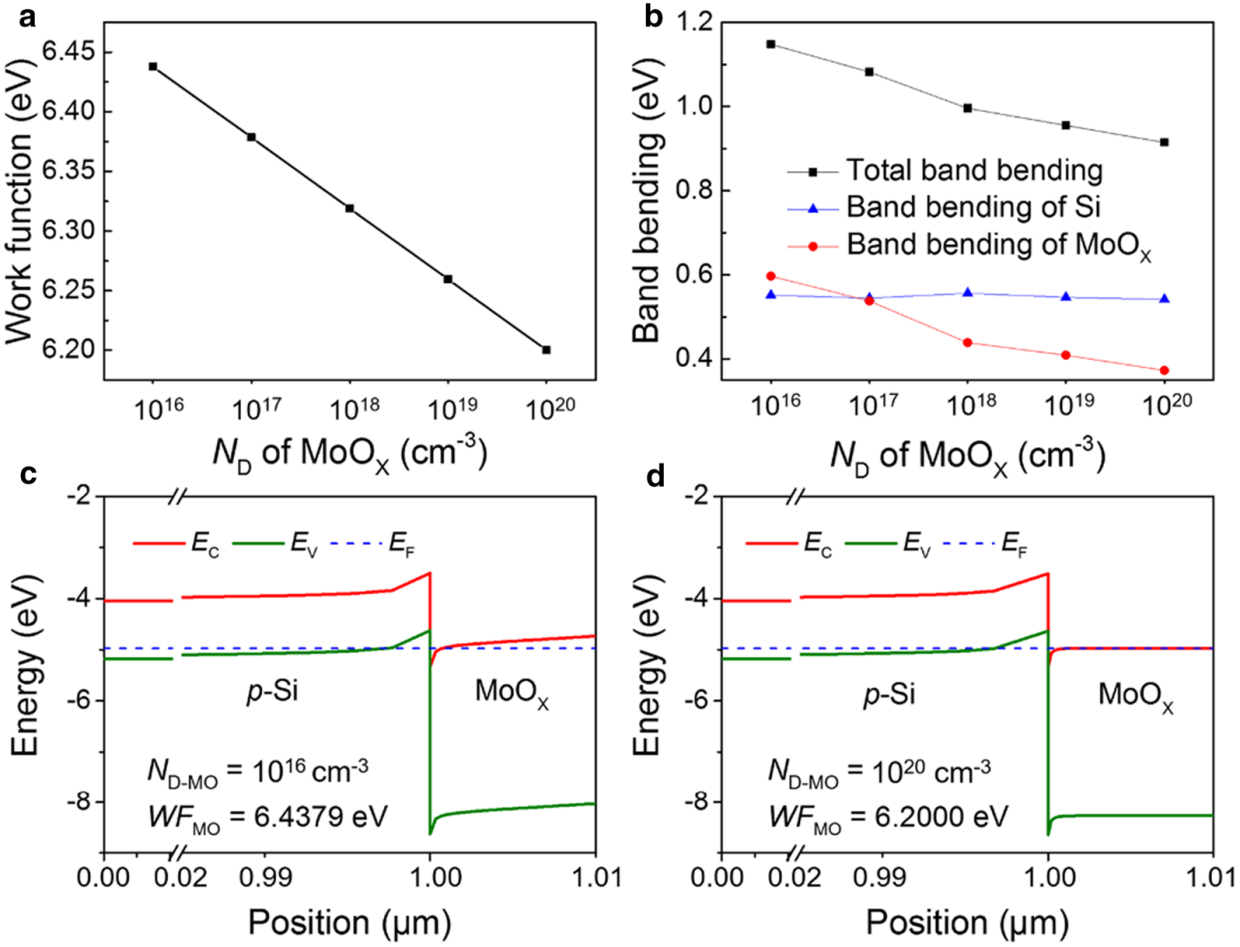
Simulated energy band results of the *p*-Si/MoO_X_ contact. **a** The relationship between the work function and *N*_D_ of MoO_X_ (*N*_D-MO_). **b** The *p*-Si, MoO_X_ and the total band bending for *p*-Si/MoO_X_ contact. The acceptor concentration of *p*-Si is 1 × 10^16^ cm^−3^. Simulated band diagrams of *p*-Si/MoO_X_ contact as the *N*_D-MO_ is **c** 1 × 10^16^ cm^−3^ and **d** 1 × 10^20^ cm^−3^, respectively

Before applying the MoO_X_ films as passivating contacts on *p*-Si wafers, one-dimensional energy band simulations are conducted using AFORS-HET [44] to get a clear image of the *p*-Si/MoO_X_ heterocontacts. The thicknesses of *p*-Si and MoO_X_ film are set as 1 μm and 10 nm, respectively. The acceptor concentration of *p*-Si is 1 × 10^16^ cm^−3^, resulting in a work function of 4.97 eV. Since MoO_X_ is an *n*-type material [53], oxygen vacancies concentration variation is simulated by changing the donor concentration at the range of 1 × 10^16^ cm^−3^ to 1 × 10^20^ cm^−3^. Figure 5a shows that the work function and donor concentration of MoO_X_ are exponentially correlated. Figure 5c, d depicts the simulated band structure as the donor concentration (*N*_D_) of MoO_X_ is 1 × 10^16^ and 1 × 10^20^ cm^−3^, respectively. Both the bands of *p*-Si and MoO_X_ are bent due to the work function difference and Fermi energy equilibrium. Efficient carrier extraction requires that photogenerated holes in the valence band of *p*-Si recombine with electrons presented in the MoO_X_ conduction band that are injected from the adjacent metal electrode [7, 54]. The band bending in *p*-Si, MoO_X_ and the total band bending are shown in Fig. 5b. As the work function of MoO_X_ (*WF*_MO_) changes, there is no obvious change in the band feature of *p*-Si. In contrast, the band bending in MoO_X_, which represents a favorable built-in electric field for electron injection, increases as its work function increases. We can conclude that the increase in the MoO_X_ work function will raise the total band bending of *p*-Si/MoO_X_ contact, most of which lies in the MoO_X_ part. Therefore, a high work function of MoO_X_ is desired from the aspect of electron injection at the *p*-Si/MoO_X_ interface.

**Fig. 6:**
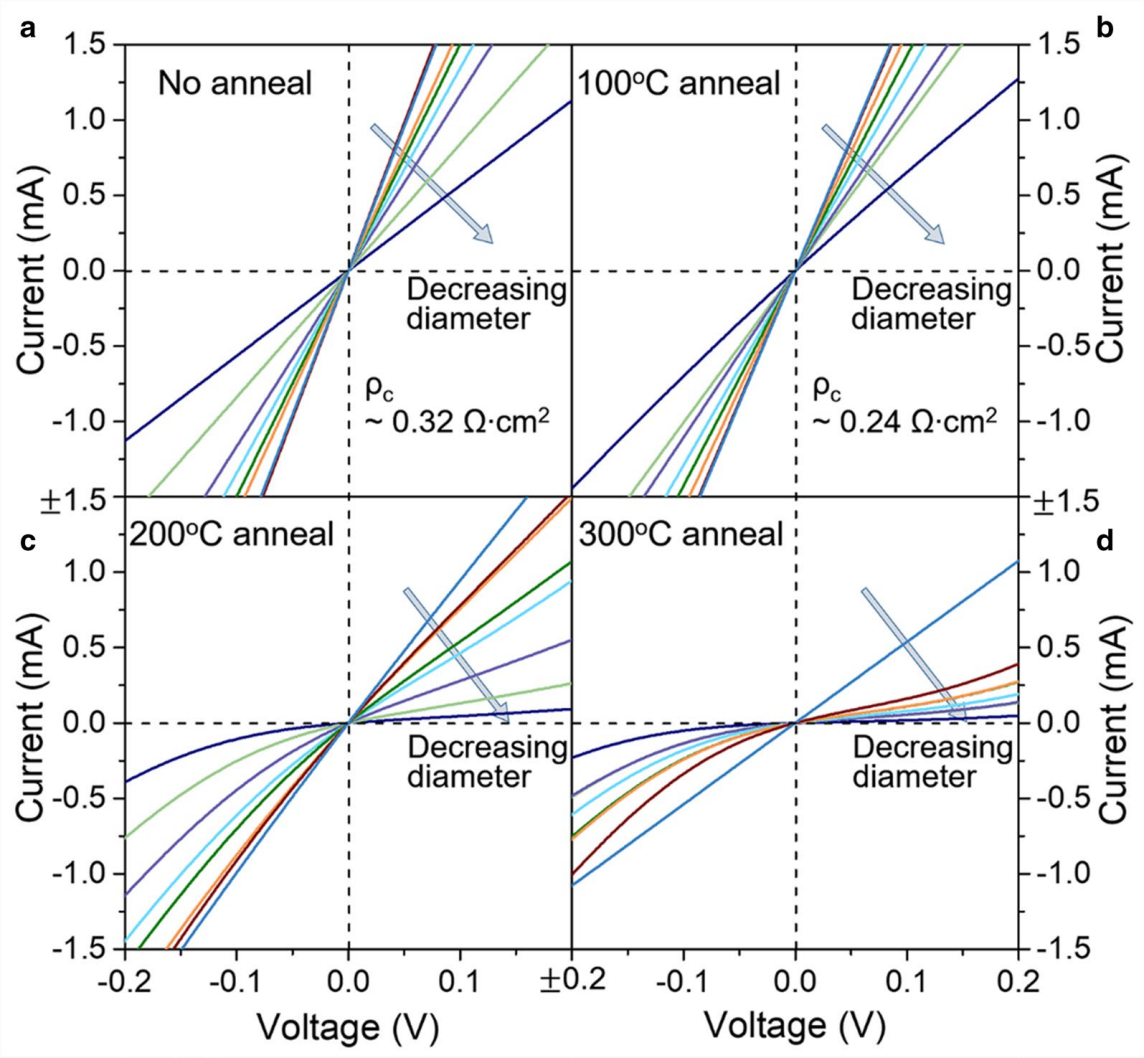
Contact resistance measurements of the 10-nm-thick MoO_X_ films on polished silicon wafers **a** without post-annealing, with post-annealing at **b** 100 °C, **c** 200 °C and **d** 300 °C

Figure 6 depicts the dark *I–V* characteristics of the *p*-Si/MoO_X_ contacts using Cox and Strack method (see Additional file 1: Figure S4 for the schematic illustration) [42]. The slope of the *I–V* curve increases with the increase of the diameter of dot electrode. The *I-V* curves of the unannealed and 100 °C-annealed samples are linear, with the specific contact resistivity (*ρ*_c_) fitted as 0.32 and 0.24 Ω‧cm^2^, respectively. Although annealing at 100 °C would make the SiO_X_ layer at the *p*-Si/MoO_X_ interface thicker, the *WF*_MO_ is higher than that of the unannealed MoO_X_ film, so the corresponding sample shows the best hole transport characteristic. The *I-V* curves of the samples annealed at 200 and 300 °C become nonlinear at small dot diameter and could not be considered as Ohmic contact. Compared with the samples annealed at 100 °C, samples annealed at higher annealing temperatures possess lower currents. As the small drop of work function, the main reason would be that higher annealing temperature causes thicker SiO_X_ layer at the *p*-Si/MoO_X_ interface, making it more difficult for carriers to tunnel through the oxide barrier.

The passivation qualities of the MoO_X_(10 nm)/*p*-Si heterojunctions as a function of thermal treatment are characterized in terms of effective minority carrier lifetime (*τ*_eff_). The injection-level-dependent *τ*_eff_s is shown in Additional file 1: Figure S5, where the *τ*_eff_s at an injection level of 1 × 10^15^ cm^−3^ are listed in Table 3. The unannealed MoO_X_ film shows the best passivation ability. Higher treating temperature leads to lower *τ*_eff_, which is the combined result of the chemical passivation of the interfacial SiO_X_ and the field effect passivation of MoO_X_, as larger X in SiO_X_ means fewer dangling bonds of silicon and larger X in MoO_X_ means larger built-in electric field intensity.

**Fig. 7:**
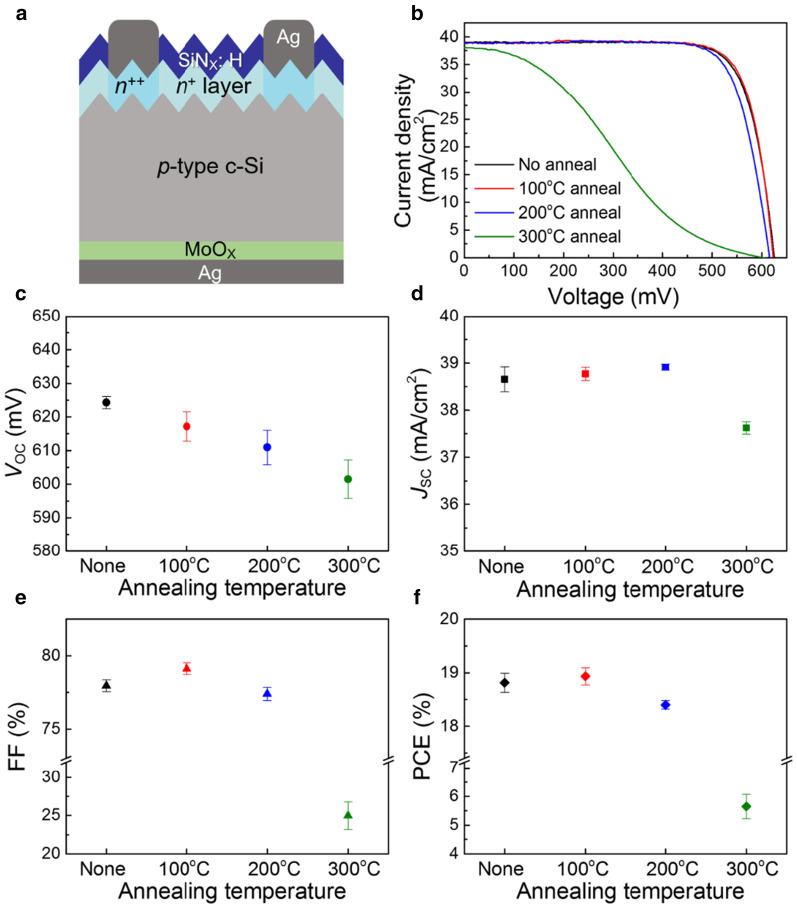
**a** Cross-sectional schematic, **b** *J–V* curves and **c–f** average *J–V* parameters of the *p*-Si/MoO_X_/Ag solar cells with MoO_X_ films annealed at different temperatures

The MoO_X_ films are then adopted into the *p*-Si/MoO_X_(10 nm)/Ag configuration (Fig. 7a) to investigate the influence of MoO_X_’s electronic properties on the device performance. The light current density versus voltage (*J–V*) curves are shown in Fig. 7b. The average *J–V* characteristics are shown in Fig. 7c–f. The lower *V*_OC_s after annealing are in line with the lower *τ*_eff_. All cells, except for the ones with MoO_X_ annealed at 300 °C, share similar *J*_SC_ (~ 38.8 mA/cm^2^), which means the minor difference in optical index of MoO_X_ and variation in the thickness of the interfacial SiO_X_ have little influence in the effective optical absorption of bulk silicon at long wavelength range. The best *PCE* of solar cells with unannealed MoO_X_ films is 18.99%, which is similar to our previous report [26]. A *PCE* of 19.19% is achieved when 100 °C annealing is applied. The *PCE* improvement mainly comes from the elevated fill factor (*FF*) with reduced series resistance, which is consistent with the low contact resistance in Fig. 6b. Inefficient transport of holes leads to the decrease of *FF*, which is prominent on the devices with 300 °C annealing. Higher annealing temperatures lead to *PCE*s drop that is originated from reduced *V*_OC_ (degraded field effect passivation of MoO_X_) and *FF* (thicker SiO_X_ interlayer reduces the carrier tunneling probability). As the MoO_X_ thin films are capped with Ag electrodes, the performance degradation could be mainly originated from the high-temperature induced elemental diffusion at the MoO_X_/Ag interface as demonstrated in the previous report [26]. The diffusion of Ag atoms into MoO_X_ will decrease MoO_X_’s work function, as the Fermi levels align at equilibrium by the transfer of electrons from metals to MoO_X_ [19, 55, 56].

Overall, the performance of the *p*-Si/MoO_X_ heterojunction solar cell is affected by the passivation quality, work function and band-to-band tunneling [34] properties of the hole-selective MoO_X_ film. The passivation performance of the present structure is still poor, leading to relatively lower *V*_OC_. Therefore, efficient surface passivation will be a research focus for nondoped carrier selective contacts.

## Conclusions

In summary, MoO_X_ films with different oxygen vacancy concentrations were obtained by post-annealing at different temperatures. The O/Mo atomic ratio of MoO_X_ films is linearly related to their work function. Compared with the intrinsic MoO_X_ film, the one annealed at 100 °C obtained less oxygen vacancy and higher work function. Energy band simulation shows that the band bending of *p*-Si in the *p*-Si/MoO_X_ contact is basically the same when the work function of MoO_X_ varies from 6.20 eV to 6.44 eV. Nevertheless, a larger work function yields increased band bending in MoO_X_ film. Experimental results indicate that the moderately improved work function of MoO_X_ annealed at 100 °C is favorable for hole selectivity. The corresponding solar cell with optimized full rear *p*-Si/MoO_X_/Ag contact achieved a *PCE* of 19.19%.

## Supplementary Information

**Additional file 1: Figure S1**. Atomic force microscopy images of the MoO_X_ thin films at different post-annealing temperatures. **Figure S2**. Green light (532 nm) Raman scattering intensity of polished Si surface and MoO_X_ films. **Figure S3**. Si 2p XPS spectra of the MoO_X_ films on Si wafers at different post-annealing temperatures. **Figure S4**. Schematic diagram of the test sample, electrode contact pattern, and test circuit for a specific contact resistivity measurement. **Figure S5**. Injection-level-dependent effective minority carrier lifetime of bare Si and MoO_X_ films at different post-annealing temperatures

## Data Availability

The datasets used and analyzed during the current study are available from the corresponding author on reasonable request.

## Abbreviations

*c*-Si: Crystalline silicon
*p*-Si: *p*-Type crystalline silicon
*n*-Si: *n*-Type *c*-Si
PCE: Power conversion efficiency
AFM: Atomic force microscope
XPS: X-ray photoelectron spectroscopy
UPS: Ultraviolet photoemission spectroscopy
QSSPC: Quasi-steady-state photo conductance
RMS: Root mean square
WF: Work function
FF: Fill factor
