# Supplementary material for: Post-annealing Effect on Optical and Electronic Properties of Thermally Evaporated MoOX Thin Films as Hole-Selective Contacts for p-Si Solar Cells
Source: Nanoscale Res Lett. 2021 May 19;16:87. doi: 10.1186/s11671-021-03544-9 (PMC8134614; doi:10.1186/s11671-021-03544-9)
Supplement: Supplementary file 1 — Additional file 1: Figure S1. Atomic force microscopy images of the MoOX thin films at different post-annealing temperatures. Figure S2. Green light (532 nm) Raman scattering intensity of polished Si surface and MoOX films. Figure S3. Si 2p XPS spectra of the MoOX films on Si wafers at different post-annealing temperatures. Figure S4. Schematic diagram of the test sample, electrode contact pattern, and test circuit for a specific contact resistivity measurement. Figure S5. Injection-level-dependent effective minority carrier lifetime of bare Si and MoOX films at different post-annealing temperatures [file 11671_2021_3544_MOESM1_ESM.docx]

**Supplementary Information**

**Post-Annealing Effect on Optical and Electronic Properties of Thermally Evaporated MoO_X_ Thin Films as Hole-selective Contacts for p-Si Solar Cells**

Yuanwei Jiang, ^a, b, §^ Shuangying Cao,^b, d, §^ Linfeng Lu,^b, d^ Guanlin Du,^b, d^ Yinyue Lin,^b, d^ Jilei Wang,^c^ Liyou Yang,^c^ Wenqing Zhu,^a,^ * Dongdong Li ^b, d,^ *

^a^ School of Materials Science and Engineering, Shanghai University, 149 Yanchang Road, Jing’an, Shanghai 200072, China

^b^ CAS Key Lab of Low-Carbon Conversion Science and Engineering, The Interdisciplinary Research Center, Shanghai Advanced Research Institute, Chinese Academy of Sciences, 99 Haike Road, Zhangjiang Hi-Tech Park, Pudong, Shanghai 201210, China

^c^ Jinneng Clean Energy Technology LTD, 533 Guang’an Street, Jinzhong 030600, China

^d^ University of Chinese Academy of Sciences, 19 Yuquan Road, Beijing 100049, China

^§^ Contributed Equally

E-mail address: [lidd@sari.ac.cn](mailto:lidd@sari.ac.cn); [wqzhu@mail.shu.ed1u.cn](mailto:wqzhu@mail.shu.edu.cn)


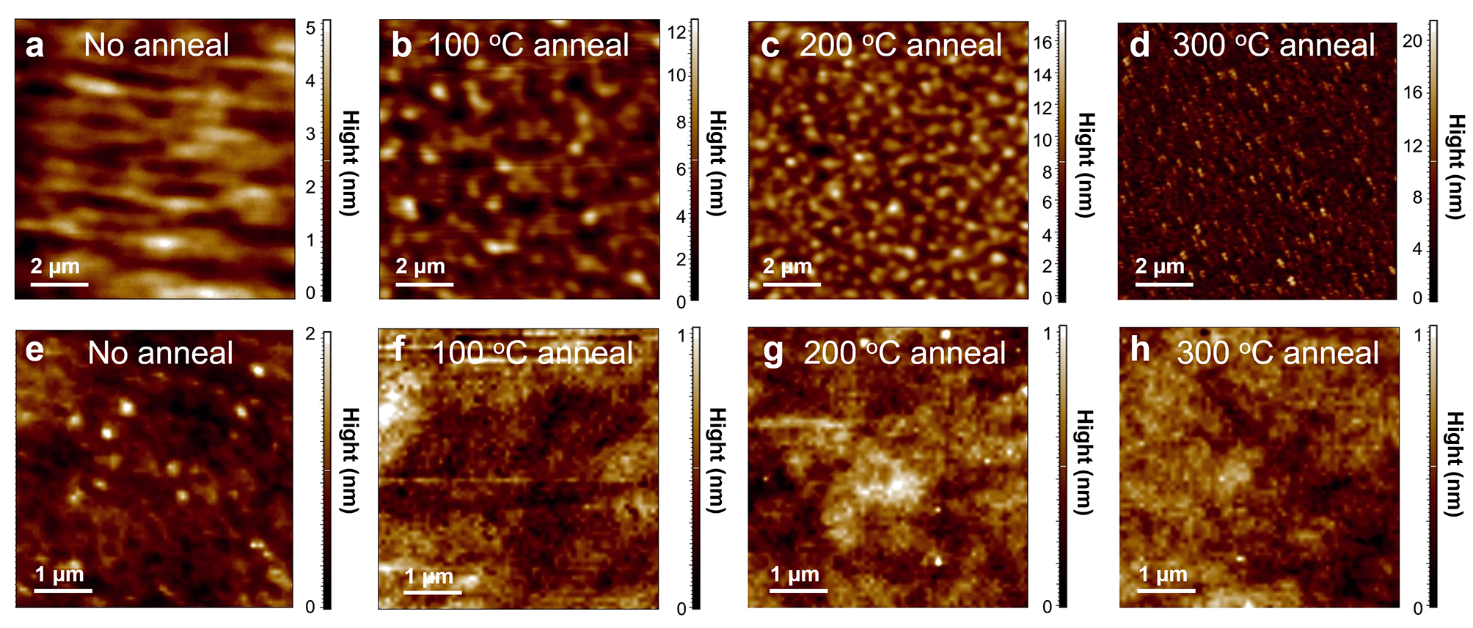


**Figure S1.** Atomic force microscopy of the (a-d) 10-nm-thick and (e-f) 20-nm-thick MoO_X_ thin films on silica glasses (a, e) without and with post annealing at (b, f) 100 ^o^C, (c, g) 200 ^o^C and (d, h) 300 ^o^C, respectively.





**Figure S2.** Green light (532 nm) Raman scattering intensity of polished silicon surface (black line) and the 20-nm-thick MoO_X_ film without annealing on silicon wafer (red line). The spectra for the annealed MoO_X_ films are not shown as they are the same as that of the unannealed one.





**Figure S3.** Si 2p XPS spectra of the 10-nm-thick MoO_X_ films on silicon wafers (a) without post annealing, with post annealing under (b) 100 ^o^C, (c) 200 ^o^C and (d) 300 ^o^C.


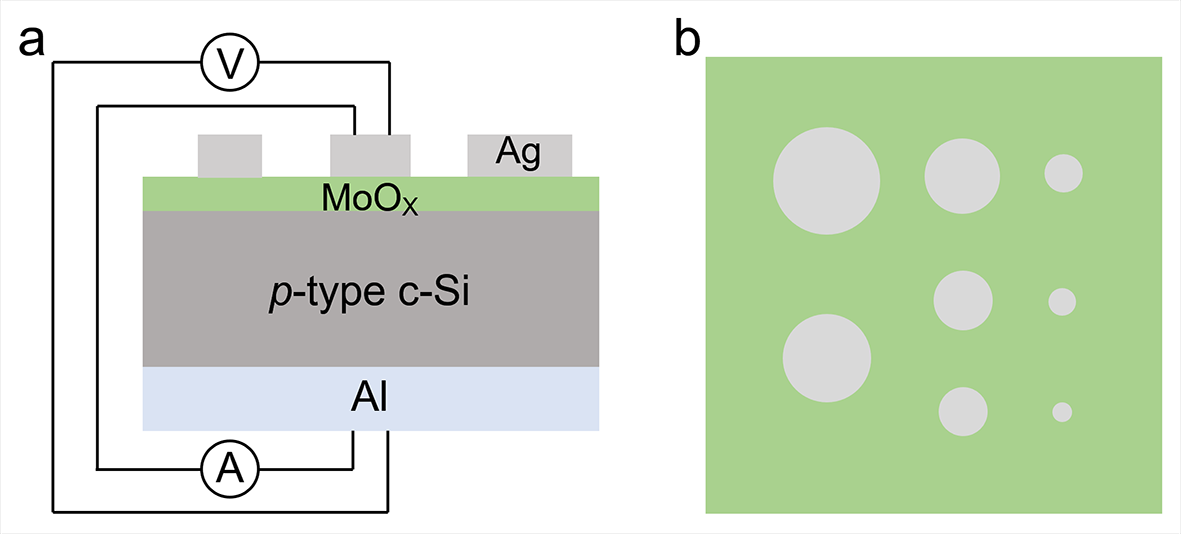


**Figure S4.** (a) Schematic diagram of the sample and test circuit for specific contact resistivity measurement. The thicknesses of Ag, MoO_X_, *p*-type c-Si and Al films are 300 nm, 10 nm, 170 μm and 1 μm, respectively. (b) Schematic diagram of the contact pattern of the test samples. The diameters of the dots are 2.4 mm, 2.0 mm, 1.8 mm, 1.6 mm, 1.4 mm, 1 mm, 0.8 mm and 0.6 mm, respectively.


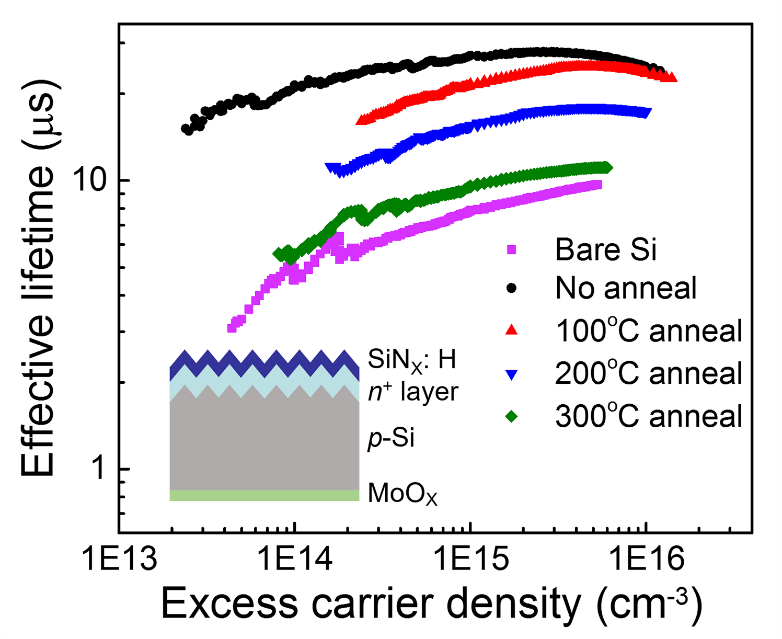


**Figure S5.** Injection-level-dependent effective minority carrier lifetime of the silicon wafers covered by MoO_X_ (10 nm) films as a function of thermal treatment. Bare Si and Si covered with MoO_X_ without annealing process are also measured as references. The inset shows the schematic diagram of the test samples.
